# Multiple Exposures Enhance Both Item Memory and Contextual Memory Over Time

**DOI:** 10.3389/fpsyg.2020.565169

**Published:** 2020-12-01

**Authors:** Haoyu Chen, Jiongjiong Yang

**Affiliations:** ^1^School of Psychological and Cognitive Sciences, Peking University, Beijing, China; ^2^Beijing Key Laboratory of Behavior and Mental Health, Peking University, Beijing, China

**Keywords:** episodic memory, repetition, contextual memory, recollection, discriminative learning

## Abstract

Repetition learning is an efficient way to enhance memory performance in our daily lives and educational practice. However, it is unclear to what extent repetition or multiple exposures modulate different types of memory over time. The inconsistent findings on it may be associated with encoding strategy. In this study, participants were presented with pairs of pictures (same, similar, and different) once (see section “Experiment 1”) or three times (see section “Experiment 2”) and were asked to make a same/similar/different judgment. By this, an elaborative encoding is more required for the “same” and “similar” conditions than the “different” condition. Then after intervals of 10 min, 1 day, and 1 week, they were asked to perform a recognition test to discriminate a repeated and a similar picture, followed by a remember/know/guess assessment and a contextual judgment. The results showed that after learning the objects three times, both item memory and contextual memory improved. Multiple exposures enhanced the hit rate for the “same” and “similar” conditions, but did not change the false alarm rate significantly. The recollection, rather than the familiarity, contributed to the repetition effect. In addition, the memory enhancement was manifested in each encoding condition and retention interval, especially for the “same” condition and at 10-min and 1-day intervals. These results clarify how repetition influences item and contextual memories during discriminative learning and suggest that multiple exposures render the details more vividly remembered and retained over time when elaborative encoding is emphasized.

## Introduction

In our everyday lives, we usually have to remember a large number of events and general knowledge. How to improve our memory ability is one of the central issues in memory research. Repetition learning is an efficient way to enhance memory performance (Ebbinghaus, 1964). Episodic memory is enhanced when an event is exposed repetitively, with detailed and vivid information remaining (Cabeza and St Jacques, 2007; Yang et al., 2016). Semantic memory could also be established after the knowledge is learned multiple times in the same or different contexts (Vargha-Khadem et al., 1997; Elward and Vargha-Khadem, 2018).

Generally, repetition learning triggers reactivation process, which results in changes in memory traces (Nadel and Moscovitch, 1997). However, how repetition learning changes memory representations is still in an intensive debate. There are two views to account for the effect of repetition learning or multiple exposures on memory. One view emphasizes that by multiple exposures, memory representation transforms from hippocampus-dependent to neocortex-dependent, thus making memory more semanticized, losing fine-grained details. For example, based on the multiple trace theory (Nadel and Moscovitch, 1997; Moscovitch et al., 2006), Yassa and Reagh (2013) further posit that each time an event is reactivated, a similar but not identical memory trace is established. The overlapping elements of the trace are assumed to become strengthened, leaving a core representation of the event (Reagh and Yassa, 2014). Hence, repetition learning leads to a more general memory with the contextual details forgotten and false memory emerging over time (Yassa and Reagh, 2013; Reagh and Yassa, 2014; Kim et al., 2019). In support of this view, the enhanced memory performance is usually shown as increased hit rates, but simultaneously increased FA rates after multiple exposures (Jacoby, 1999; Poppenk et al., 2010; Reagh and Yassa, 2014; Reagh et al., 2017). In an investigation by Reagh and Yassa (2014), after participants learned a series of objects that were presented once or three times, they were asked to make an old/new judgment for the old, similar, and new objects. The results showed that general recognition was enhanced, but the ability to discriminate similar from new objects was diminished (in moderate similarity) after three exposures.

The other view is that multiple exposures not only lead to a generalized memory representation, but also enhance detailed memory by facilitating elaborative encoding. The enhanced memory performance is shown as increased hit rates and decreased FA rate (e.g., Yang et al., 2016; McCormick-Huhn et al., 2018). In addition, the underlying processes may differentially contribute to the repetition effect. According to the dual process model of recognition memory, both recollection and familiarity processes contribute to discriminating between old and new items during test (Yonelinas and Jacoby, 1995; Tulving, 2002; Yonelinas, 2002; Eichenbaum et al., 2007; but see Dunn, 2004; Williams et al., 2013). To support the elaborative view, some studies have suggested that multiple exposures lead to enhanced recollection contribution, hence increased ability to discriminate between targets and lures (e.g., Yang et al., 2016; McCormick-Huhn et al., 2018). The familiarity process remained relatively stable (e.g., Yang et al., 2016) or greater for older adults (McCormick-Huhn et al., 2018). For example, in a study by McCormick-Huhn et al. (2018), participants learned single words from different categories once or twice. Then, recognition memory (old, lures from the same category, and new words) was tested by a remember/know/new task 24 h later. The results showed that compared to learning once, repetition learning in either the same or different context led to significant memory enhancement, and the repetition effect was contributed by the recollection process.

One main difference between the two views is whether detailed memory is established after repetition. The inconsistent findings on it may be associated with encoding strategy. Studies have confirmed that different encoding tasks have significant effects on subsequent memory performance (Craik and Lockhart, 1972; Craik, 2002). Deeper or more elaborative encoding is associated with higher levels of memory retention. For example, when participants were asked to pay attention to specific parts of perceptual features of an object during encoding, distinctive features of each picture were elaboratively processed, and subsequent old/new recognition performance was improved (Koutstaal et al., 1999). The difference in encoding strategy may also influence the effect of repetitive learning. When the encoding strategy is elaborative, repetitive learning enables participants to have more chance to deeply process the stimuli, leading to higher subsequent memory performance including detailed information. In this study, we adopted a discriminative learning paradigm to test this possibility.

The discriminative learning paradigm has been suggested to be an efficient way for elaborative processing. In this paradigm (Zhou et al., 2018), two similar (e.g., two dog pictures) or different (e.g., one dog and one flower pictures) objects were presented simultaneously, and participants were asked to make a similar/different judgment. So in this case, discriminative learning refers to a process to distinguish between two pictures, whether they belong to the same or different concepts. During test, they were presented with an old and a similar picture and performed a two-alternative forced choice (2AFC) task. If they judged the picture was old, they further decided under what encoding condition (similar or different) the picture was learned (i.e., contextual memory; Zhou et al., 2018). The results showed that the objects in the “similar” condition were better remembered in terms of both item details and their contextual features than in the “different” condition. When eye movements were measured, more saccades between the two similar objects during encoding predicted higher item and contextual memory performance in the “similar” condition. It suggests that discriminating between the “similar” objects triggers more elaborative encoding to facilitate subsequent item and contextual memories.

The important feature of discriminative learning is that item memory is enhanced in both detailed and general aspects when two similar objects are compared. After discriminative learning, Chen et al. (2019) adopted a recognition test during which participants were presented an old picture or a new but similar picture and thereby requested to make an old/new judgment. The results showed that the hit rate and FA rate were both higher for the “similar” than “different” condition. As similar objects were used as lures in the test, the participants had to discriminate between the old and the similar pictures; memory after discriminative learning reflects a detailed representation. On the other hand, when participants have difficulty in discriminating lure pictures from old ones in terms of details, but still have memory of an object’s concept, they judge them as “old.” So, a higher FA rate indicates a more gist-based memory representation (Reagh et al., 2017; Lee et al., 2018). As discriminative learning of similar objects enhanced both detailed and gist-based memory representations, and both item and contextual memories, this paradigm is appropriate to explore how different types of memory change after repetitive exposures.

In addition, when participants discriminated similar objects only once, their enhanced item and contextual memories retained until 1 week (Zhou et al., 2018; Chen et al., 2019). But there are intensive debates on whether multiple exposures produce slower forgetting of subsequent memory (e.g., Slamecka and McElree, 1983; Bogartz, 1990; Gardiner and Java, 1991; Hockley and Consoli, 1999; Yang et al., 2016). For example, Slamecka and McElree (1983) showed that learning three times led to greater memory performance of words and word pairs than learning once, but the forgetting rate remained stable from immediately to 5 days after the test. Other studies showed that learning multiple times led to slower forgetting at shorter intervals, which was mainly contributed by the recollection process (e.g., Yang et al., 2016). Whether multiple exposures influence forgetting rate after discriminative learning needs to be clarified. Memory forgetting is usually measured as the interaction between retention interval and other factors (e.g., Slamecka and McElree, 1983; Gardiner and Java, 1991; Hockley and Consoli, 1999). However, this interaction may be influenced by initial memory performance. So the forgetting rate should be measured when the initial performance was controlled.

In summary, we applied the discriminative learning paradigm (Zhou et al., 2018) to explore the effect of multiple exposures on subsequent memory over time. During encoding, two groups of participants learned the object pairs once (see section “Experiment 1”) or three times (see section “Experiment 2”) by making a same/similar/different judgment. Then, after the intervals of 10 min, 1 day, and 1 week, their item memory [followed by a remember/know/guess (RKG) judgment] and contextual memory were tested. In addition to the “similar” and “different” conditions, the “same” condition was included. The three conditions differed in the requirement of elaborative encoding. In the “different” condition, the two objects were conceptually different, so elaborative processing of detailed information was not necessary to make a “different” response. In the “same” and “similar” condition, the two objects shared the same concept, so elaborative encoding was required to discriminate between similar objects or make sure that the two objects were exactly the same. The three intervals were included to explore whether the repetition effect after discriminative learning could remain with the passage of time. The forgetting rate was calculated for each condition by controlling the initial memory performance.

The aforementioned two views have different predictions for the effect of repetition on discriminative learning on critical parameters such as hit/FA rates and recollection/familiarity contributions. Based on the view of generalized representation, when participants discriminated objects multiple times, a more stable semantic representation of the object is established, leading to higher FA rates and greater familiarity contribution, and the contextual memory should not be improved. As memory representation is more semanticized and more stable, memory forgetting should be slower after repetition. Instead, based on the view of elaborative encoding, multiple exposures lead to greater memory for the details related to the objects and their contexts, especially for the “same” condition. Accordingly, the recollection contribution is enhanced. In this case, memory representations decay over time, and the effect of multiple exposures should not influence memory forgetting significantly.

## Experiment 1

### Materials and Methods

#### Participants

Twenty-five healthy, right-handed participants (eight males) with a mean age of 20.80 ± 2.22 years were recruited in section “Experiment 1.” The overall sample size for the experiment was based on an *a priori* power analysis (G*Power 3.1.9.6; University of Kiel, Germany) and previous studies (e.g., Yang et al., 2016; Zhou et al., 2018). In order to obtain adequate power (i.e., *α* = 0.05, 1 − *β* = 0.95) and detect moderate effect size (i.e., *f* = 0.25) for the interaction of encoding condition (3) and retention interval (3), we would need a total sample of at least 22 participants for each learning group. All of the participants were native Chinese speakers, and they all provided written informed consent in accordance with the procedures and protocols approved by the Review Board of School of Psychological and Cognitive Science, Peking University.

#### Materials

Two within-subjects factors were included in the study: encoding (same, similar, and different) and retention interval (10 min, 1 day, and 1 week).

Seven hundred twenty objects (240 triplets) were selected from Hemera Photo Clipart and the Internet. Each triplet included three similar color pictures with the same basic concept/name (e.g., dog, tomato). The three pictures differed in dimensions such as shape, color, orientation, and number. They were in the same size of 640 × 480 pixels and with the white background. The 720 pictures were rated by a group of 23 participants (12 males, mean age of 22.83 ± 2.67 years) who did not participate in the experiments. The participants named the pictures and rated their familiarity (i.e., how familiar they felt the object were. One for most familiar, five for most unfamiliar) and similarity within the triplets (i.e., how similar the two pictures were. One for most dissimilar, five for most similar). As one concept triplet had three similar pictures, three similarity rating scores for every two pictures of a triplet were acquired and averaged as one similarity score for each concept (Zhou et al., 2018; Chen et al., 2019). The naming accuracy for the pictures was 0.91 ± 0.12, the familiarity score was 1.81 ± 0.33, and the similarity score was 2.93 ± 0.51.

All triplets were first randomly assigned to four groups (Groups A–D), with one group used for the “same” condition, one for the “similar” condition, and the other two for the “different” condition (Chen et al., 2019). Next, each group was assigned to three different sets (S1, S2, and S3) for three retention intervals. The three pictures within a triplet were differentially used during encoding and test. For the same condition (A1-A1), one picture within a triplet was learned during encoding and presented as the old picture during test; one of the other two pictures was randomly used as the lure picture during test (A2 or A3). For the similar condition (B1-B2), two pictures within a triplet were paired during encoding; one of them was randomly used as the old picture during test, and the third picture was used as the lure picture (B3). For the different condition (C1-D1), one picture in each of the two groups was randomly paired during encoding; one of them was used as the old picture during the test, and the similar picture to the other picture was used as the lure picture (C2 or D2). The materials in groups and sets were counterbalanced (*p* > 0.60) so that each picture had the same chance to be used in each condition.

#### Procedure

**Figure 1:**
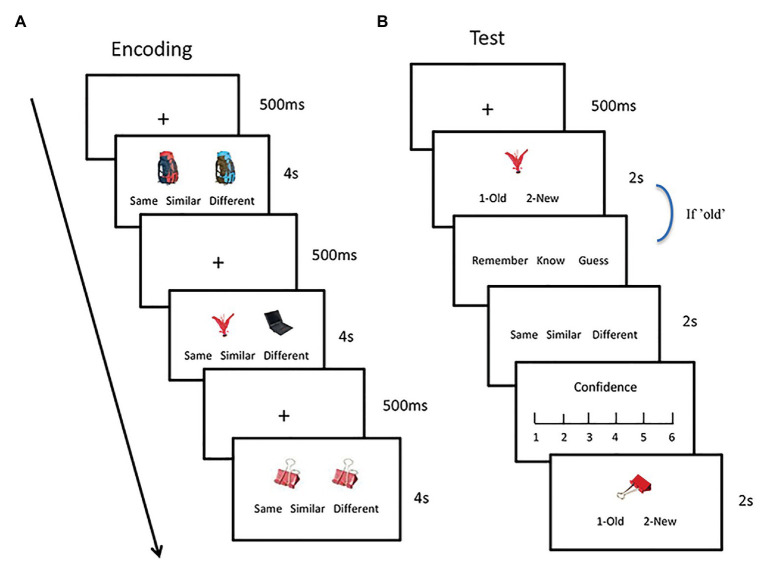
Experimental procedure. During the encoding phase, participants were asked to judge whether the two pictures were the same, similar, or different **(A)**. During the test phase, the participants finished an old/new recognition task. If the judgment was “old,” they further make a remember/know/guess and a contextual judgment task **(B)**.

During the encoding phase (Figure 1A), each picture pair was presented on the screen for 4 s, and the participants judged whether the two pictures were the same, similar, or different. All the pairs were pseudorandomly presented during the encoding phase so that no more than three stimuli that were in the same condition were presented consecutively. The position of the target/old pictures and the order of the three buttons were counterbalanced across the participants.

During the test phase, each picture was presented on the screen for 2 s, and the participants performed an old/new recognition (i.e., item memory) test and a contextual memory test. During the item memory test, they judged whether the picture was old or new as accurately and quickly as possible (Figure 1B). Half of the pictures were old, and the other half were new but similar to the old ones (i.e., lures). If the picture was judged as “old,” the participants made an RKG assessment and a contextual judgment. They responded as “remember” (R) if they could retrieve stimulus-related details. They responded as “know” (K) if they only felt that the picture was familiar without any detailed information. They responded as “guess” if they did not retrieve the stimulus by the two aforementioned processes. During the contextual judgment task, the participants determined whether the picture appeared in the same/similar/or different condition, followed by the confidence rating. The old and new pictures were pseudorandomly presented at each retention interval so that no more than three pictures in the same condition were presented consecutively.

The software we used for the presentation of the stimuli and the recording of the participants’ responses was MATLAB and its free set Psychophysics Toolbox-3 (MathWorks Co.). The participants learned the 180 pairs once and then performed the item memory and contextual memory tests at three retention intervals (60 objects per interval, 20 pairs per encoding condition). Before each test phase, to avoid a rehearsal from the study phase, the participants were asked to count backward by seven continuously from 1,000 for 5 min. The participants had separate opportunities to practice encoding and test trials before the formal phases. In particular, to ensure that they followed the instruction of the RKG procedure, they specifically practiced this part with feedback from experimenters.

#### Data Analysis

The hit rate, FA rate, corrected recognition (hit-FA), and the accuracy of contextual memory were analyzed using a repeated-measures analysis of variance (ANOVA) with retention interval (10 min, 1 day, and 1 week) and encoding condition (same, similar, and different) as within-subjects factors by the SPSS software. The accuracy of the contextual memory was calculated as the correct number of contextual judgment trials out of the total number of trials in each condition (Chen et al., 2019). The forgetting rate was estimated by the interaction between the retention interval and encoding condition (Slamecka and McElree, 1983; Gardiner and Java, 1991; Hockley and Consoli, 1999). As the results of the corrected recognition and *d*′ value were similar, and those of the contextual memory with all trials and high-confidence trials were similar, only the previous ones were reported. Partial *η*^2^ was calculated to estimate the effect size of each analysis. *Post hoc* pairwise comparisons were Bonferroni-corrected (*p* < 0.05, two-tailed).

Recollection and familiarity processes were estimated using the independent K (IRK) procedure (Yonelinas and Jacoby, 1995; Yonelinas, 2002), in which R responses are assumed to estimate recollection, whereas familiarity is estimated as the proportion of K responses divided by the proportion of non-R responses. By this, the recollection and familiarity are not only mutually exclusive, but also independently estimated. Therefore, R and IRK responses were corrected with the FA rate: recollection = *p*(R, hit) − *p*(R, FA); familiarity = [*p*(K, hit)/1 − *p*(R, hit) – *p*(K,FA)/1 − *p*(R,FA)]. Repeated-measures ANOVA tests were applied separately for recollection and familiarity processes with encoding condition and retention interval as within-subjects factors (*p* < 0.05, two-tailed). Partial *η*^2^ was calculated to estimate the effect size of each analysis. *Post hoc* pairwise comparisons were Bonferroni-corrected (*p* < 0.05, two-tailed).

### Results

During the encoding phase, the participants judged the pairs of objects accurately (0.97 ± 0.04) and quickly (1.31 ± 0.21 s). The effect of encoding condition was not significant for accuracy [*F*(2,48) = 2.75, *p* = 0.11, *η*^2^ = 0.10] but significant for reaction times [RTs; *F*(2,48) = 16.27, *p* < 0.001, *η*^2^ = 0.40]. This was because the “different” pairs were judged as the quickest (1.12 ± 0.19 s), and the “same” pairs were judged as the slowest (1.48 ± 0.38 s; *p* < 0.001).

**Figure 2:**
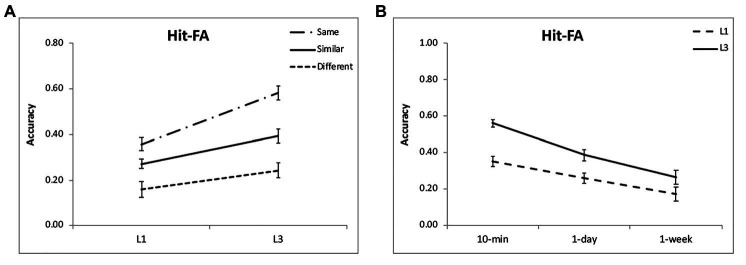
Results of the corrected recognition. Multiple exposures enhanced item memory **(A,B)** for each condition, with the greatest enhancement for the “same” condition and at intervals of 10 min and 1 day. The accuracies were averaged for different intervals **(A)** and for different encoding condition **(B)** to illustrate the interactions of group and condition, and group and interval. The error bars represent the standard errors of the means.

For the corrected recognition, there was a significant effect of encoding condition [*F*(2,48) = 26.76, *p* < 0.001, *η*^2^ = 0.53; Figure 2A]. Further analysis showed that memory performance was the highest for the “same” condition, then the “similar” condition, and the lowest for the “different” condition (*p* < 0.001; Table 1). Besides, memory accuracy decreased over time [*F*(2,48) = 16.40, *p* < 0.001, *η*^2^ = 0.41; Figure 2B], but the interaction between condition and time interval was not significant [*F*(4,96) = 1.41, *p* = 0.24, *η*^2^ = 0.06]. The results suggest that after discriminative learning of “same” and “similar” pictures, the memory performance remained at a high level over time.

**Table 1:** Results for group L1.

		10 min			1 day			1 week		
		Same	Similar	Diff	Same	Similar	Diff	Same	Similar	Diff
Hit-FA	Mean	0.49	0.33	0.24	0.35	0.28	0.15	0.24	0.20	0.09
	SD	0.23	0.22	0.24	0.20	0.13	0.16	0.19	0.20	0.13
Hit	Mean	0.77	0.75	0.51	0.64	0.70	0.43	0.49	0.49	0.31
	SD	0.14	0.10	0.17	0.15	0.15	0.17	0.19	0.19	0.20
FA	Mean	0.28	0.42	0.27	0.30	0.41	0.28	0.25	0.29	0.22
	SD	0.15	0.18	0.15	0.17	0.15	0.18	0.15	0.16	0.18
Contextual memory	Mean	0.54	0.65	0.34	0.29	0.55	0.21	0.14	0.31	0.11
	SD	0.22	0.13	0.21	0.18	0.17	0.15	0.11	0.16	0.09
Recollection	Mean	0.38	0.28	0.14	0.20	0.18	0.05	0.05	0.06	0.00
	SD	0.23	0.18	0.15	0.17	0.14	0.09	0.08	0.09	0.03
Familiarity	Mean	0.25	0.10	0.11	0.19	0.14	0.04	0.15	0.15	0.05
	SD	0.20	0.19	0.20	0.19	0.16	0.12	0.20	0.15	0.09

**Figure 3:**
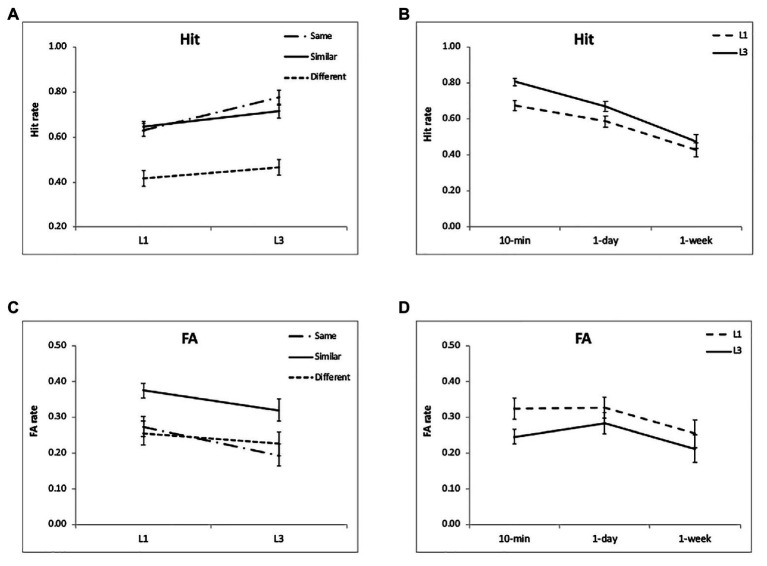
Results of the hit and FA rates. Multiple exposures significantly increased the hit rate for the “same” and “similar” conditions **(A)** and kept the FA rate relatively stable **(C)**. There were no significant interactions between group and retention interval for the hit and FA rates **(B,D)**. The accuracies were averaged for different intervals **(A,C)** and for different encoding condition **(B,D)** to illustrate the interactions of group and condition, and group and interval. The error bars represent the standard errors of the means.

The hit rate had a significant effect of encoding condition [*F*(2,48) = 72.28, *p* < 0.001, *η*^2^ = 0.75; Figure 3A]. The hit rates for the “same” and “similar” conditions were higher than that for the “different” condition (*p* < 0.005), but they did not differ significantly (*p* = 0.51; Table 1). There was also a significant effect of encoding condition for the FA [*F*(2,48) = 27.39, *p* < 0.001, *η*^2^ = 0.53], showing that the FA rates of the “same” and “different” conditions were lower than that of the “similar” condition (*p* < 0.005), but they did not differ significantly (*p* = 0.31; Table 1; Figure 3C). Both the hit rate [*F*(2,48) = 40.27, *p* < 0.001, *η*^2^ = 0.63] and FA rate [*F*(2,48) = 6.20, *p* = 0.004, *η*^2^ = 0.21] decreased significantly over time (Figures 3B,D). The interactions between condition and time interval were not significant for the hit rate [*F*(4,96) = 1.33, *p* = 0.27, *η*^2^ = 0.05] and FA rate [*F*(4,96) = 1.43, *p* = 0.23, *η*^2^ = 0.06]. These results suggest that discriminating “similar” pictures leads to both higher hit rate and higher FA rate.

**Figure 4:**
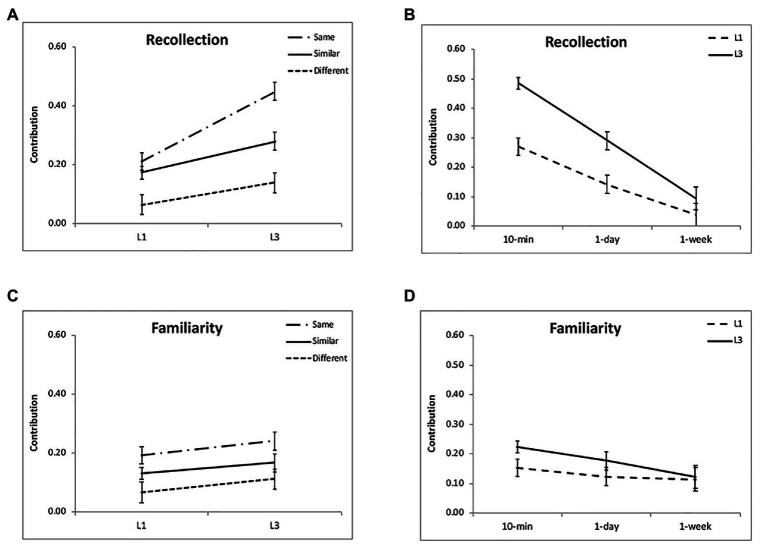
Results of recollection and familiarity contributions. Multiple exposures enhanced the recollection process, especially for the “same” condition **(A)** and at 10-min and 1-day intervals **(B)**. The interactions between group and encoding condition and between group and retention interval were not significant for the familiarity contribution **(C,D)**. The accuracies were averaged for different intervals **(A,C)** and for different encoding condition **(B,D)** to illustrate the interactions of group and condition, and group and interval. The error bars represent the standard errors of the means.

Regarding the contribution of recollection, there was a significant effect of encoding condition [*F*(2,48) = 25.58, *p* < 0.001, *η*^2^ = 0.54; Figure 4A] and a significant interaction between condition and time interval [*F*(4,96) = 6.28, *p* < 0.001, *η*^2^ = 0.21; Table 1]. Further analysis showed that its contribution was higher for the “same” and “similar” conditions than the “different” condition (*p* < 0.001), but they did not differ significantly (*p* = 0.10) except at 10 min (*p* = 0.01). Regarding the contribution of familiarity, there was a significant effect of encoding condition [*F*(2,48) = 8.14, *p* = 0.001, *η*^2^ = 0.25; Figure 4C]. Further analysis showed that its contribution was higher for the “same” and “similar” conditions than the “different” condition (*p* < 0.05), but the “same” and “similar” conditions did not differ (*p* = 0.10). The effect of time interval [*F*(2,48) = 1.16, *p* = 0.32, *η*^2^ = 0.05; Figure 4D] and the interaction were not significant [*F*(4,96) = 1.90, *p* = 0.12, *η*^2^ = 0.07]. Both the recollection and familiarity contributions were significantly higher than chance level (0) for each condition (*p* < 0.05) except the “different” condition at 1 week for the recollection (*p* = 0.52; Table 1; Figures 4A,B). The proportion of guess response did not show significant effects of retention interval [*F*(2,48) = 0.74, *p* = 0.48, *η*^2^ = 0.03] or encoding condition [*F*(2,48) = 0.87, *p* = 0.42, *η*^2^ = 0.04]. The results suggest that after discriminative learning, both recollection and familiarity contribute to the enhanced memory under the “same” and “similar” conditions over time.

**Figure 5:**
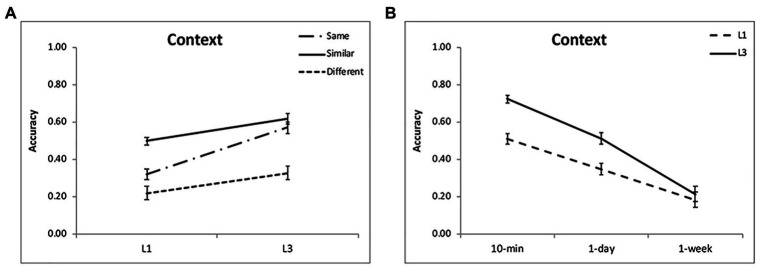
Results of contextual memory. Multiple exposures enhanced contextual memory **(A,B)** for each condition, with the greatest enhancement for the “same” condition and at intervals of 10 min and 1 day. The accuracies were averaged for different intervals **(A)** and for different encoding condition **(B)** to illustrate the interactions of group and condition, and group and interval. The error bars represent the standard errors of the means.

For contextual memory, the effect of encoding condition was significant [*F*(2,48) = 40.47, *p* < 0.001, *η*^2^ = 0.63], showing that the source memory for the “similar” condition was the highest, then the “same” condition, and lowest for the “different” condition (Table 1; Figure 5A). The accuracy decreased over time [*F*(2,48) = 76.20, *p* < 0.001, *η*^2^ = 0.76; Figure 5B]. There was a significant interaction between time interval and encoding [*F*(4,96) = 5.64, *p* = 0.001, *η*^2^ = 0.20], as the difference between the “same” and “similar” condition was larger with the passage of time (*p* < 0.002). The contextual memory for the “same” condition was higher than that for the “different” condition at 10-min interval (*p* < 0.001), but they were comparable at 1-day and 1-week intervals (*p* > 0.30; Table 1). It suggests that discriminative learning of similar objects significantly enhances the contextual memory, and this remains over time. Although the picture in the “same” condition was well recognized, the contextual memory was lower than that in the “similar” condition and comparable to that in the “different” condition.

Overall, the main result of section “Experiment 1” was that the recognition memory for pictures was greater after the two same or similar pictures were learned together than when the two different pictures were together. The contextual memory was higher for the “similar” than for the other conditions and remained over time. Thus, after discriminating between “similar” objects, the memories for both the objects and the contexts were significantly improved. Discriminating between the “same” objects significantly enhanced item memory only. The result of section “Experiment 1” also showed the RTs during encoding were significantly slower for the “same” and “similar” conditions than for the “different” condition, and memory enhancement relied on both recollection and familiarity processes. It suggests that discriminating same and similar objects facilitates elaborative encoding process, which was consistent with Zhou et al. (2018) when the 2AFC task was employed. We further investigated the effect of multiple exposures on memory of item and contextual information in section “Experiment 2”.

## Experiment 2

### Materials and Methods

#### Participants

Twenty-five healthy, right-handed participants (eight males) with a mean age of 21.76 ± 1.92 years were recruited in section “Experiment 2.” The sample size was determined by power analysis using G*Power 3.1.9.6 (University of Kiel, Germany) and referred to previous studies (e.g., Yang et al., 2016; Zhou et al., 2018). A prior power analysis revealed that a total sample size of at least 22 participants would provide 95% power to detect effects. All of the participants were native Chinese speakers, and they all provided written informed consent in accordance with the procedures and protocols approved by the Review Board of School of Psychological and Cognitive Science, Peking University.

#### Materials and Procedures

The materials and procedures were the same as those in section “Experiment 1,” except that the participants learned the picture pairs three times. The pairs were presented in a block-wise manner; i.e., all the picture pairs were presented once, and then they were presented for the second and third times. The orders of the pairs in three presentations were different for each participant.

#### Data Analysis

Data analysis was the same as that in section “Experiment 1.” In addition, we compared the parameters when learning once and three times. The ANOVAs were performed with group (once, three times) as between-subjects factor and encoding (same, similar, and different) and time interval (10 min, 1 day, and 1 week) as within-subjects factors. In addition, to control for the initial memory performance, the forgetting rate was calculated for each condition as follows: (memory at 10 min − memory at 1 week)/(memory at min). A repeated-measures ANOVA was conducted with group and encoding condition as within-subjects factors for both item and contextual memories. *Post hoc* pairwise comparisons were Bonferroni-corrected (*p* < 0.05, two-tailed).

### Results

#### Learning Three Times

During the encoding phase, the participants judged the object pairs accurately (0.98 ± 0.01) and quickly (1.34 ± 0.28 s). The effect of encoding condition was significant for accuracy [*F*(2,48) = 3.38, *p* = 0.04, *η*^2^ = 0.13] and RTs [*F*(2,48) = 13.81 *p* < 0.001, *η*^2^ = 0.37]. This was because the “different” pairs were judged as the quickest (1.19 ± 0.25 s) and most accurate (0.99 ± 0.01), and the “same” pairs were judged as the slowest (1.51 ± 0.43 s) and least accurate (0.98 ± 0.02; *p* < 0.001).

For the corrected recognition, there was a significant effect of encoding condition [*F*(2,48) = 95.21, *p* < 0.001, *η*^2^ = 0.80; Figure 2A] and a significant interaction [*F*(4,96) = 2.79, *p* = 0.03, *η*^2^ = 0.10; Table 2]. Besides, memory accuracy decreased over time [*F*(2,48) = 43.07, *p* < 0.001, *η*^2^ = 0.64; Figure 2B]. As the case in section “Experiment 1,” memory performance was the highest for the “same” condition, then the “similar” condition, and the lowest for the “different” condition (*p* < 0.001; Table 2).

**Table 2:** Results for group L3.

		10 min			1 day			1 week		
		Same	Similar	Diff	Same	Similar	Diff	Same	Similar	Diff
Hit-FA	Mean	0.79	0.51	0.39	0.55	0.38	0.23	0.40	0.29	0.11
	SD	0.14	0.19	0.22	0.16	0.20	0.16	0.16	0.13	0.17
Hit	Mean	0.93	0.83	0.66	0.79	0.76	0.46	0.61	0.55	0.27
	SD	0.06	0.12	0.16	0.14	0.14	0.21	0.19	0.19	0.13
FA	Mean	0.14	0.33	0.27	0.24	0.38	0.23	0.21	0.26	0.18
	SD	0.13	0.15	0.15	0.16	0.18	0.14	0.14	0.14	0.12
Contextual memory	Mean	0.85	0.73	0.59	0.61	0.62	0.30	0.25	0.30	0.10
	SD	0.11	0.19	0.19	0.17	0.19	0.18	0.20	0.20	0.11
Recollection	Mean	0.76	0.44	0.27	0.43	0.30	0.13	0.16	0.10	0.02
	SD	0.14	0.23	0.18	0.17	0.18	0.12	0.14	0.12	0.05
Familiarity	Mean	0.29	0.19	0.19	0.26	0.17	0.10	0.18	0.15	0.04
	SD	0.31	0.22	0.15	0.22	0.21	0.13	0.15	0.16	0.08

For the “hit” rate, there was a significant effect of encoding condition [*F*(2,48) = 155.77, *p* < 0.001, *η*^2^ = 0.87; Figure 3A]. The hit rate for the “same” condition was significantly higher than that in the “similar” and “different” conditions (*p* < 0.01; Table 2). For the FA rate, there was a significant effect of encoding condition [*F*(2,48) = 24.25, *p* < 0.001, *η*^2^ = 0.50; Figure 3C]. The FA rate in the “similar” conditions was higher than that in the “same” and “different” conditions (*p*'s < 0.05). This pattern was similar to that in section “Experiment 1.” Both the hit rate [*F*(2,48) = 57.85, *p* < 0.001, *η*^2^ = 0.71] and FA rate [*F*(2,48) = 4.26, *p* = 0.02, *η*^2^ = 0.15] changed significantly over time (Figures 3B,D). The interaction between condition and time interval was significant for the hit rate [*F*(4,96) = 3.33, *p* = 0.01, *η*^2^ = 0.12] and FA rate [*F*(4,96) = 7.28, *p* < 0.001, *η*^2^ = 0.23], because the hit rate of the “same” condition was higher than that of the “similar” condition only at 10 min (*p* < 0.001), and the FA rate of the “same” conditions was higher than that of the “different” condition at 10 min (*p* < 0.001), but they were comparable for the two conditions at 1-day and 1-week (Table 2).

Regarding the contribution of recollection, there was a significant effect of encoding condition [*F*(2,48) = 84.04, *p* < 0.001, *η*^2^ = 0.78] and a significant interaction [*F*(4,96) = 15.06, *p* < 0.001, *η*^2^ = 0.39; Figure 4A]. This was because the difference between the “same” and “similar” condition was the largest at 10-min, then decreased over time, and comparable at 1-week (*p* = 0.33; Table 2). Regarding the contribution of familiarity, there was a significant effect of encoding condition [*F*(2,48) = 10.48, *p* < 0.001, *η*^2^ = 0.30; Figure 4C]. The effect of time interval [*F*(2,48) = 4.75, *p* = 0.01, *η*^2^ = 0.15; Figure 4D] and the interaction were not significant [*F*(4,96) = 1.03, *p* = 0.40, *η*^2^ = 0.04]. Further analysis showed that its contribution was the greatest for the “same” condition, then the “similar” condition, and least for the “different” condition (*p* < 0.05). The proportion of guess response increased over time [*F*(2,48) = 7.40, *p* = 0.002, *η*^2^ = 0.24], but did not show significant effect of encoding condition [*F*(2,48) = 0.64, *p* = 0.53, *η*^2^ = 0.03]. The results suggest that the both the recollection and familiarity processes contribute to the enhanced memory after discriminative learning, especially for the “same” and “similar” condition.

For contextual memory, the effect of encoding condition was significant [*F*(2,48) = 46.33, *p* < 0.001, *η*^2^ = 0.66; Figure 5A], showing that the contextual memory for the “same” and “similar” conditions were comparable (*p* = 0.52) but both were higher than that for the “different” condition (*p* < 0.001; Table 2). There was a significant interaction between time interval and encoding [*F*(4,96) = 8.81, *p* < 0.0011, *η*^2^ = 0.27]. This was because the contextual memory for the “same” condition was greater than that for the “similar” condition at 10-min interval (*p* = 0.01) and was comparable to that for the “similar” condition afterwards (*p* = 1.0).

Overall, the patterns of the corrected recognition, hit rate, FA rate, contribution of recollection and familiarity were generally similar to those in section “Experiment 1,” except that the difference between the “same” and “similar” conditions was significant for the hit rate, contributions of recollection and familiarity. The contextual memory for the “same” condition was significantly enhanced.

### Comparison of Experiments 1 and 2

**Figure 6:**
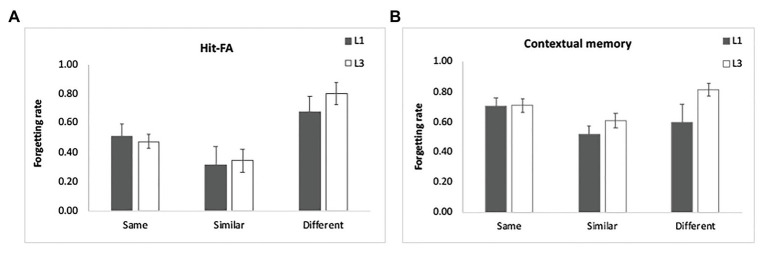
Results of the forgetting rate. Multiple exposures did not significantly influence the forgetting of item memory **(A)** and contextual memory **(B)**. The forgetting rates were estimated by controlling the initial memory performance for each condition. The error bars represent the standard errors of the means.

For the corrected recognition, the ANOVA results showed a significant group effect [*F*(1,48) = 21.11, *p* < 0.001, *η*^2^ = 0.31]. In addition, there was a significant interaction between group and encoding condition [*F*(2,96) = 7.85, *p* = 0.001, *η*^2^ = 0.14]. Further analysis demonstrated that the “same” condition benefitted from multiple exposures most evidently (*p* < 0.001), then the “similar” condition (*p* = 0.003) and least for the “different” condition (*p* = 0.04; Figure 2A). The interaction between group and interval was also significant [*F*(2,96) = 3.75, *p* = 0.03, *η*^2^ = 0.07], showing that learning three times led to better memory performance for each interval, but most pronouncedly for 10-min and 1-day intervals (*p* < 0.001; Figure 2B). These findings suggest that the “same” condition benefits more from multiple exposures, and the enhancement is obvious at shorter intervals. The results of the forgetting rate showed a significant effect of condition [*F*(2,96) = 13.54, *p* < 0.001, *η*^2^ = 0.23; Figure 6A], with slower forgetting for the “same” and “similar” conditions than for the “different” condition (*p* < 0.002). But the effect of group [*F*(1,48) = 0.37, *p* = 0.55, *η*^2^ = 0.008] and the interaction [*F*(2,96) = 0.25, *p* = 0.78, *η*^2^ = 0.006] was not significant. It suggests that multiple exposures do not influence forgetting of item memory.

Regarding the hit rate, the group effect was significant [*F*(1,48) = 8.28, *p* = 0.006, *η*^2^ = 0.15]. In addition, there was a significant interaction between group and encoding condition [*F*(2,96) = 6.29, *p* = 0.003, *η*^2^ = 0.12]. Multiple exposures significantly increased the hit rate for the “same” (*p* < 0.001) and “similar” (*p* = 0.04) conditions, with no significant change for the “different” condition (*p* = 0.19; Figure 3A). The interaction of group and retention interval was not significant [*F*(2,96) = 2.04, *p* = 0.14, *η*^2^ = 0.04; Figure 3B]. Regarding the FA rate, the group effect was not significant [*F*(1,48) = 2.94, *p* = 0.10, *η*^2^ = 0.06]. In addition, there was no significant interaction between group and encoding condition [*F*(2,96) = 1.84, *p* = 0.16, *η*^2^ = 0.04] or between group and retention interval [*F*(2,96) = 0.77, *p* = 0.46, *η*^2^ = 0.02; Figures 3C,D]. The results suggest that the hit rate is enhanced after multiple exposures for the “same” and “similar” conditions, but the FA rate remains relatively stable after multiple exposures.

Regarding the contribution of recollection, there was a significant interaction between group and encoding condition [*F*(2,96) = 15.32, *p* < 0.001, *η*^2^ = 0.24; Figure 4A], and between group and interval [*F*(2,96) = 9.58, *p* = 0.001, *η*^2^ = 0.17; Figure 4B]. The greater contribution of recollection for L3 than L1 was most obvious for the “same” condition and the 10-min and 1-day intervals, although group contrasts were all significant (*p* < 0.01). Regarding the contribution of familiarity, it was comparable for L1 and L3 [*F*(1,48) = 2.55, *p* = 0.12, *η*^2^ = 0.05]. In addition, there was no significant interaction between group and encoding condition [*F*(2,96) = 0.06, *p* = 0.94, *η*^2^ = 0.001] or between group and retention interval [*F*(2,96) = 1.21, *p* = 0.30, *η*^2^ = 0.03; Figures 4C,D]. These results suggest multiple exposures enhance the recollection process rather than familiarity.

For contextual memory, there was a significant interaction between group and encoding condition [*F*(2,96) = 11.40, *p* < 0.001, *η*^2^ = 0.19] and between group and retention interval [*F*(2,96) = 12.05, *p* < 0.001, *η*^2^ = 0.20]. Multiple exposures enhanced contextual memory for each condition, with the greatest enhancement for the “same” condition (Figure 5A) and for the intervals of 10 min and 1 day (*p* < 0.05; Figure 5B). There was a significant three-way interaction [*F*(4,192) = 3.55, *p* = 0.008, *η*^2^ = 0.07]. The enhancement for the “similar” condition was obvious at each interval after L1 (*p* < 0.05) and disappeared at 1 day and 1 week after L3 when compared to the “same” condition (*p* > 0.50). This indicated that discriminative learning of similar objects significantly enhances contextual memory when the stimuli were learned once. Multiple exposures increased the contextual memory especially for the “same” condition. The results of the forgetting rate showed a significant effect of condition [*F*(2,96) = 4.66, *p* = 0.01, *η*^2^ = 0.09; Figure 6B], with slower forgetting for the “similar” condition than for the “same” and “different” conditions (*p* < 0.05). But the effect of group [*F*(1,48) = 2.04, *p* = 0.16, *η*^2^ = 0.05] and the interaction [*F*(2,96) = 2.19, *p* = 0.12, *η*^2^ = 0.05] was not significant. It suggests that multiple exposures do not significantly influence forgetting of contextual memory.

## Discussion

In this study, we investigated how multiple exposures modulated item memory and contextual memory over time after discriminative learning. There were three main findings. First, after learning three times, both item memory and contextual memory performance increased. Second, the hit rate for the “same” and “similar” conditions was enhanced after multiple exposures, but the FA rate did not change significantly. In addition, the recollection contributed to the repetition effect, with no significant change for the familiarity contribution. Third, memory enhancement was manifested in each encoding condition and for all retention intervals, especially for the “same” condition and at 10-min and 1-day intervals. These results suggest that through multiple discriminative learning, the stimuli are more elaboratively encoded, making the details and contexts more vividly remembered and retained over time.

### Enhanced Memory and Multiple Exposures

Previous studies have posited two possible findings for the effect of repetition learning on subsequent memory. On the one hand, repetition learning decreases the capacity to discriminate between targets and lures, leading to enhanced general memory but impaired detailed memory (e.g., Reagh and Yassa, 2014; Reagh et al., 2017). On the other hand, repetition learning enhances elaborative encoding and hence the recollection process, leading to improved subsequent memory for details (e.g., Koutstaal et al., 1999; Yang et al., 2016; McCormick-Huhn et al., 2018). The results of our study supported the second view. The enhanced memory was manifested in both item and contextual memories. In the work herein, after learning three times, the increased discrimination ability for item memory was based on increased hit rate and relatively stable FA rate. Previous studies have shown that item-specific memory was enhanced, and false recognition was reduced when participants were asked to notice perceptual features of pictures during encoding (Koutstaal et al., 1999). Similarly, discriminative learning of similar objects enabled participants to adopt elaborative processing to compare detailed information between the objects to make a judgment (Zhou et al., 2018). By learning three times, the participants have more chance to deliberately compare the two objects, and the differences between the two objects are strengthened. In contrast, the FA rate remained relatively stable after multiple exposures. Although the FA rate was higher for the “similar” condition than the other two when learning once, it did not change significantly by learning three times. It is possible that general memory for the concept is quickly acquired and stabilized by one exposure of two similar objects; thus, more exposures are not necessary. Therefore, more exposures facilitate elaborative encoding of detailed information, which in turn leads to enhanced hit rate and memory performance for pictures.

Consistent with the change of hit and FA rates, our finding showed that higher item memory after repetition was more contributed by the recollection process rather than the familiarity. As the participants had to distinguish between the old and similar picture, the item memory reflected memory for detailed information. The detailed item memory after multiple exposures had more vivid subjective feeling and perceptual information (McCormick-Huhn et al., 2018). This supported the finding that the hit rate increased after repetition. The elaborative encoding after multiple exposures facilitated the participants remembering more detailed information about the objects, thereby rendering the recollection process more of a contributor. In contrast, there was no significant group effect or group-related interactions for the familiarity process, suggesting that the enhanced memory is not contributed by familiar feeling of the objects.

In addition to item memory, the results demonstrated that contextual memory was also enhanced. During the retrieval phase, the participants were asked to judge the condition (same/similar/different) where the objects were learned. Although the contextual memory is associated with the recollection process (Davachi et al., 2003; Davachi, 2006; Eichenbaum et al., 2007), it differs from item memory in that the contextual memory relies more on information related to spatial or temporal sources of the object rather than detailed information of the object itself. Thus, by multiple exposures, the relations between objects and their contexts are enhanced significantly.

Although our findings supported the view of elaborative processing, it does not mean that the generalization view is not correct. It is possible that core content and details of the memory are selectively strengthened and connected (Nadel and Moscovitch, 1997), but variable contextual details associated with each reactivation of the memory are weakened (Yassa and Reagh, 2013). We consider that the encoding task is important for the effect of repetition on subsequent memory. For example, Reagh and Yassa (2014) showed that the FA rate was greater when the pictures were presented three times rather than just once. They asked participants to make an indoor/outdoor judgment for single objects. In contrast, discriminative learning emphasizes the relationship between the two pictures, especially when the pictures were the same or similar. The encoding difference may render memory for detailed information more likely distinctive and contextualized after discriminative learning, and the distinctive representations are reactivated and strengthened after multiple exposures. Future studies are required to include both single pictures and picture pairs in a study to clarify the boundary conditions for different effects of repetition learning. These two effects may be mediated by different brain mechanisms (Wagner et al., 2000; Manelis et al., 2013; Kremers et al., 2014; Chen et al., 2019). In addition, different from Reagh and Yassa (2014), in which they included old, similar, and new objects during test, we only included the old and similar objects to ensure the participants focus on the detailed memory without having the strategic change through the test (Brainerd and Reyna, 1993, 2015). It is interesting to include new pictures during test to assess gist/conceptual memory in addition to detailed/perceptual memory at the same time.

### Discriminative Learning and Multiple Exposures

One important feature of discriminative learning was that elaborative encoding is required to discriminate between two objects for the “same” and “similar” conditions. In section “Experiment 1,” the item memory in the “same” and “similar” condition was higher than that in the “different” condition. Multiple exposures enhanced the performance of item and contextual memories as well as recollection process most in the “same” condition. The longer encoding time indicated that discriminating between the “same” objects takes more time than between the “similar” and “different” objects. Although the two pictures were the same under the “same” condition, the participants had to adopt elaborative strategy and process every detail to ensure that the two objects were the same. So, the “same” condition was analog to the “similar” condition but required more elaborative processing. Multiple exposures of same objects enabled the participants to have more chance to elaborately process the detailed and contextual information, which led to higher item and contextual memories.

After the participants learned the similar objects once, both the hit rate and FA rate were higher than the pairs in the “different” condition. It suggests that presenting two similar objects of a concept facilitate the general memory of the concept, in addition to an enhanced detailed memory for the objects (Chen et al., 2019). Multiple exposures enhanced the item and contextual memories for the “similar” condition, although the effect was smaller than those for the “same” condition. Note that the FA rate was higher for the “similar” condition irrespective of repetition. This made the corrected recognition lower for the “similar” (vs. “same”) condition. In addition, the contextual memory was higher for the “similar” than the “same” condition after learning once, whereas the difference disappeared after learning three times. So the advantage of adopting “similar” (vs. “same”) condition was that the enhanced memory effects appeared right after learning once, especially for the contextual memory. It suggests that discriminative learning of similar objects is efficient to quickly improve memory in both details and contexts, and repetition is not necessary.

### Retention Interval and Multiple Exposures

The enhanced memories of item and context for the “similar” condition over the “different” condition were shown from 10 min to 1 week. Multiple exposures further enhanced memories at shorter intervals. There was a significant interaction between group and retention interval for item memory and contextual memory, showing that the group difference was significant at each interval but more obvious at 10 min and 1 day. With repetition, the stimuli are more elaboratively processed, which leads to more stable memory representations (Xue et al., 2011) and higher memory accuracy over time (Litman and Davachi, 2008; Yang et al., 2016). The higher enhancement at shorter intervals may be mainly because of massed learning mode (Cepeda et al., 2006; Mazza et al., 2016). In this study, the objects were presented repetitively in three blocks, which was a typical manipulation of massed learning (Cepeda et al., 2006). Previous studies have also found that compared to distributed learning, massed learning enhances recent memory significantly, whereas distributed learning improved associative memory at longer intervals (Litman and Davachi, 2008; Yang et al., 2016).

On the other hand, as memory performance increased after repetition, it is necessary to control the initial memory (at 10-min interval) to clarify the effect of multiple exposures on memory forgetting. The results showed the forgetting rates of item and contextual memories had no significant group effects, or interactions between group and encoding condition. The results thus suggest that multiple exposures at encoding do not modulate subsequent memory consolidation process (Slamecka and McElree, 1983). Although memory performance in each condition declined from 10 min to 1 week, multiple exposures did not change the forgetting pattern. The significant interaction between group and interaction for hit-FA was mainly due to higher memory accuracy, rather than stronger memory consolidation process.

The influence of multiple exposures on memory performance has important practical implications. Memory impairments are common in elderly people and patients with brain lesion. Some people with memory impairments, such as patients with amnesia and severely deficient autobiographical memory, are characteristic of the deficits in encoding processes (Palombo et al., 2016, 2018). As multiple exposures enhanced elaborative encoding, repetitive learning could be used as an efficient way to improve memory retention for memory-impaired patients (e.g., Green et al., 2014). Future studies with neuroimaging investigations could also help to clarify to what extent the hippocampus and cortical regions (Santangelo et al., 2018, 2020) are involved in enhanced encoding after repetition for patients with memory deficits.

Furthermore, the decline was contributed by the recollection process, shown as a significant interaction between retention interval and group for the recollection rather than for the familiarity. The results supported the view that contribution of recollection is associated with memory forgetting over time. As proposed by Sadeh et al. (2014), memories relying on recollection are more sensitive to decay but are relatively resistant to interference from irrelevant information (Gardiner and Java, 1991; Hockley and Consoli, 1999; Yang et al., 2016). Through multiple exposures, the recollection contribution significant increased, making the forgetting rate slower.

The RKG procedure is widely used to estimate the underlying processes during recognition (Tulving, 1985; Donaldson, 1996; Yonelinas, 2002; Wixted and Stretch, 2004). Some may argue that the distinction of recollection and familiarity reflect the difference of strong and weak memory and confidence experience (Dunn, 2004; Squire et al., 2007; Williams et al., 2013). According to this proposal, forgetting is a change from stronger to weaker memory representation, and multiple exposures lead to stronger memory. If so, the recollection process should increase by repetition and decrease by longer intervals, whereas the familiarity should decrease by repetition and increase with the passage of time. Contrary to the prediction, we found that the familiarity remained unchanged for L1 and L3 and decreased over time. The distinction in recollection and familiarity well explained the current findings on forgetting and learning effect. We therefore suggest that the recollection/familiarity distinction is an appropriate way to account for the underlying process of recognition in this study.

Although detailed memory is susceptible to be forgotten rapidly (e.g., Tuckey and Brewer, 2003; Huebner and Gegenfurtner, 2012; Andermane and Bowers, 2015; Sekeres et al., 2016), through discriminative learning of similar objects, some detailed memory and contextual memory remained at 1 week. This pattern was observed for both L1 and L3 conditions. The transformation trace theory model states that with the passage of time, memory representation could have both gist and detailed forms, and they can be transformed in certain conditions (Winocur and Moscovitch, 2011; Moscovitch et al., 2016; Robin and Moscovitch, 2017). In addition, the recollection contribution decreases more rapidly than the familiarity (Gardiner and Java, 1991; Hockley and Consoli, 1999; Sadeh et al., 2014; Yang et al., 2016). Thus, at 1 week, both the recollection and familiar contributions helped the participants make correct judgments to discriminate between the old and lure objects.

## Conclusion

After learning three times, both item memory and contextual memory performance increased over time. The enhanced item memory was shown as higher hit rate rather than the FA rate, and with more of a contribution of the recollection process rather than the familiarity. In addition, multiple exposures enhanced the memory performance especially for the “same” condition and at 10-min and 1-day intervals. Overall, these results suggest that when elaborative processing is emphasized during encoding, multiple exposures enable recollection more pronouncedly, rendering the details and contexts more vividly remembered and retained over time. Therefore, the strategies combining elaborative encoding and multiple exposures could apply to elderly adults and patients with brain lesion who have memory impairments and help them improve memory abilities over time.

## Data Availability Statement

The raw data supporting the conclusions of this article will be made available by the authors, without undue reservation.

## Ethics Statement

The studies involving human participants were reviewed and approved by the Ethics Committee of School of Psychological and Cognitive Sciences, Peking University. Participants received written and oral information of the study before they gave their written consent. The patients/participants provided their written informed consent to participate in this study.

## Author Contributions

HC designed and performed the research, and analyzed the data. JY designed the research, analyzed the data, and wrote the paper. All authors contributed to the article and approved the submitted version.

### Conflict of Interest

The authors declare that the research was conducted in the absence of any commercial or financial relationships that could be construed as a potential conflict of interest.
